# Effects of YouTube Health Videos on Mental Health Literacy in Adolescents and Teachers: Randomized Controlled Trial

**DOI:** 10.2196/76004

**Published:** 2025-07-31

**Authors:** Rebekka Schröder, Tim Hamer, Victoria Kruzewitz, Ellen Busch, Ralf Suhr, Lars König

**Affiliations:** 1 Stiftung Gesundheitswissen Berlin Germany; 2 Charité – Universitätsmedizin Berlin Berlin Germany

**Keywords:** adolescents, mental health, mental health literacy, health literacy, schools, teachers, students, YouTube Health videos, RCT

## Abstract

**Background:**

Adolescence is a critical period for mental health development, yet prevalences of mental health problems are high among young people. Enhancing mental health literacy in school settings could be an effective strategy for the promotion of mental well-being and prevention of mental health struggles. One promising approach to achieving this goal involves equipping both students and teachers with accessible multimedia resources—such as YouTube Health videos—to enhance their mental health literacy.

**Objective:**

The study evaluates the effectiveness of a short educational YouTube Health video for promoting mental health literacy in adolescents and teachers.

**Methods:**

Two independent samples of 352 adolescents and 502 teachers from Germany were recruited from a large panel, representative of the German population with internet access. Participants of each sample were allocated to an experimental group (176 adolescents and 254 teachers) and a control group (176 adolescents and 248 teachers) through randomization. The experimental group watched a YouTube Health video designed to increase mental health literacy, while the control group watched a video similar in style but on a different topic. Before and after watching the publicly available YouTube Health videos, mental health knowledge was assessed as a primary outcome through topic-specific quizzes and a self-report in a web-based survey. In addition, all participants were asked to rate the educational, visual, and overall quality of the YouTube Health videos and their usability in school settings. The primary hypotheses were tested with ANOVAs. The quality and usability items were analyzed descriptively.

**Results:**

For the adolescents, there were significant main effects of time (*F*_1,350_=46.34, *P<*.001, η^2^_p_=0.117) and group (*F*_1,350_=6.05, *P=*.01, η^2^_p_=0.017) and a significant time×group interaction (*F*_1,350_=39.15, *P<*.001, η^2^_p_=0.101) on stress-specific knowledge, indicating a higher increase in knowledge in the experimental group than in the control group. Similarly, for teachers, significant main effects of time (*F*_1,500_=107.31, *P<*.001, η^2^_p_=0.177) and group (*F*_1,500_=58.07, *P<*.001, η^2^_p_=0.104) and a significant time×group interaction (*F*_1,500_=82.59, *P<*.001, η^2^_p_=0.142) were found. The same pattern of results was observed for the knowledge self-reports in both the students (time: *F*_1,347_=103.65, *P<*.001, η^2^_p_=0.230; group: *F*_1,347_=8.59, *P=*.004, η^2^_p_=0.024; time×group interaction: *F*_1,347_=29.11, *P<*.001, η^2^_p_=0.077) and teachers (time: *F*_1,500_=115.40, *P<*.001, η^2^_p_=0.188; group: *F*_1,500_=41.16, *P<*.001, η^2^_p_=0.076; time×group interaction: *F*_1,500_=64.24, *P<*.001, η^2^_p_=0.114). Overall, the educational, visual, and overall quality of the videos and their usability in school settings were rated as positive by both adolescents and teachers.

**Conclusions:**

The study findings demonstrate that short educational YouTube Health videos are effective tools for the promotion of mental health literacy among both students and their teachers. Overall, this evaluation paves the way for a wider implementation of mental health education in schools in order to create a more supportive and informed environment to promote mental well-being.

**Trial Registration:**

German Clinical Trial Register DRKS00036854; https://drks.de/search/en/trial/DRKS00036854/details

## Introduction

Mental health problems are among the leading causes of disability worldwide, especially in young people [1-3]. Approximately 30% of the German adult population are estimated to have mental disorders [4], a concerning problem also observed in younger generations: In school-aged children and adolescents, approximately 20% report mental health problems, with a slight decrease before the COVID-19 pandemic [5] and a pronounced rise since then [6]. Furthermore, recent studies have shown that signs of depression [7] and eating disorders [8] are especially prevalent among children and adolescents. Given the high prevalence of mental health problems among students and the significant amount of time students spend in school, schools have been identified as critical settings for the promotion of mental health and well-being among children and adolescents [9,10]. In this context, teachers take on multiple roles related to mental health support for their students, including monitoring of student status, referral to other service providers, and teaching behavioral interventions [11]. At the same time, teachers are also affected by personal mental health problems and report high levels of work-related stress [12-14], which makes them not only a provider but also a potential recipient of mental health support.

One promising approach to improving mental health is by focusing on mental health literacy [15,16]. Building on the concept of general health literacy, mental health literacy is defined as “understanding how to obtain and maintain positive mental health; understanding mental disorders and their treatments; decreasing stigma related to mental disorders; and enhancing help-seeking efficacy” [16]. An important facet of mental health literacy is mental health knowledge, which is considered to be an essential prerequisite for enabling individuals to take practical action to improve their own mental well-being and that of others [15,17]. For example, lack of knowledge has been identified as a critical barrier to mental health care access in young people [18]. To date, there is little evidence concerning mental health literacy levels among adolescents, partly due to a lack of broad measuring tools for this target group [19,20]. In a recent systematic review in low- and middle-income countries, low levels of recognition and knowledge about mental health problems were observed in children and adolescents [19]. In the general population in Germany, intermediate mental health literacy levels were delineated, with marked interindividual differences [21].

In adults, positive associations of both mental and general health literacy and lower stigma against mental disorders and more positive attitudes toward psychotherapeutic treatment have been observed [22-26]. Similarly, in adolescents, higher mental health literacy has been found to correlate with help-seeking attitudes and intentions [27]. In addition, mental health literacy has been identified as an important determinant of mental well-being in schools [28] but not consistently with general well-being in adolescence [27]. There is tentative evidence that universal prevention interventions may improve mental health literacy but with overall low quality of evidence [29], which is why further research in this area is warranted.

Despite their unique role, schools are not fully equipped to support students’ mental health, and many teachers report insufficient knowledge, confidence, and resources to do so [11,30-34]. In addition, there are mixed results concerning teachers’ mental health literacy levels, which highlights that they might benefit from more targeted support [34,35].

In the past, video-based interventions were found to be effective tools to enhance various components of mental health literacy, including knowledge [36]. At the same time, in a recent systematic review, the quality of many publicly available health-related videos on YouTube was found to be low, and a need for more high-quality content was identified [37]. With the aim of supporting general and mental health literacy across different target groups, the independent nonprofit foundation Stiftung Gesundheitswissen previously conceptualized and produced short educational videos in various health-related fields for target groups of the general population and adolescents. The videos are presented on a YouTube channel that is part of the YouTube Health program. This programs flags health-related content from authoritative and credible sources and makes content from these channels more easily accessible in searches on health-related topics [38]. Channels eligible for this program need to fulfill a set of standards including adhering to principles of health-related information sharing [39] and employing licensed health professionals that oversee and review the channels’ content.

Specifically, to support mental health literacy, several YouTube Health videos covering the topic of stress were developed recently. This study focuses on one of these videos that gives a general introduction on the topic. The video addresses the first facet of mental health literacy, namely understanding how to obtain and maintain positive mental health [16], and is directed at both adolescents and adults from the general population. The primary aim of this investigation was to evaluate this stress-related YouTube Health video as a proof-of-concept study to demonstrate that short educational videos of this type can promote mental health knowledge as a facet of mental health literacy. The video was compared with a similar YouTube Health video on a different topic in a randomized pre-post control group design in 2 independent samples of school-aged students and general education teachers. Significant differences in stress-specific knowledge between the experimental group and control group were expected after, but not before, watching the respective YouTube Health video (primary hypothesis of this study). The secondary aim was to examine the overall quality, educational value, visual appeal, and usability of the YouTube Health videos for their use and dissemination in school settings. Importantly, for these factors, no differences between the experimental and the control videos were hypothesized, as the videos were designed in a similar way and only differed in content. Therefore, their overall quality, educational quality, and visual appeal were not tested for group differences.

## Methods

### Ethical Considerations

A study protocol including detailed information on the study procedures, measures, and data security was submitted to the ethics committee of the Berlin Medical Association, which did not raise any professional or ethical objections against the study (reference: Eth-42/23). All participants were informed about the study procedures and aims. Specifically, participants were informed that the study aimed to investigate the extent to which explanatory videos can be used to promote health literacy and to examine their quality. They were asked for their consent via checkboxes in a web-based questionnaire before data collection. For the students, informed consent was provided by a parent or next of kin. Participants could withdraw from the study at any given time without any negative consequences. Participants were compensated with the standard compensation scheme of the panel (for details about the panel, see the Recruitment and Data Collection section). They received bonus points that can be exchanged for vouchers, cashed out, or donated. Anonymized and complete data sets were provided to the foundation by the market research institute responsible for data collection.

This study was part of a larger research project that evaluated several short educational videos produced by Stiftung Gesundheitswissen and assessed health literacy and further health-related constructs in 3 independent samples of the general population, teachers, and students. Each of these samples was divided into 4 groups (ie, 4 video interventions) through randomization (for more details on randomization, see the Procedures section). For this study, only 2 samples (teachers, students) and 2 groups (stress video, fake news video) are relevant, as they focus on the topic of mental health literacy in school settings. This study therefore does not cover the primary outcome of the larger research project, namely digital health literacy. The larger research project was retrospectively registered at the German Clinical Trial Register (DRKS), the German World Health Organization (WHO) primary register, with the registration number DRKS00036854. Further publications focusing on different topics will likely arise from this project.

### Sample

#### Recruitment and Data Collection

Data were collected with the help of the *forsa* (Gesellschaft für Sozialforschung und statistische Analysen mBH) market research institute. Participants were recruited in November 2023 and December 2023 from the forsa.omninet panel. The forsa.omninet panel is a large panel with more than 100,000 participants representative of the population with internet access and sufficient knowledge of the German language in Germany. Participants for the panel are recruited via telephone to ensure that rare internet users are also represented. It is not possible to apply for the panel, which means that a single person cannot participate with multiple accounts. Two independent samples were recruited: students aged 14 years to 18 years and teachers. The information, if the panelists were teachers, was available to the market research institute due to prior surveys and was confirmed by the panelists before data collection. For the students, initial recruitment took place via their parents. In the first step, parents of potential students within the relevant age group were invited. If they confirmed they had at least one child in the relevant age group and provided informed consent for them to participate in the study, data collection was initiated with their child. If they had more than one child, the child with the most recent birthday was selected for study participation. All participants were invited to take part in the study via email, with a maximum of 2 reminders sent.

#### Inclusion and Exclusion Criteria

Inclusion criteria for both samples were sufficient knowledge of the German language and internet access (guaranteed for all panelists through the recruitment strategy of the panel). In addition, students had to be between 14 years and 18 years old, and teachers had to currently work as a teacher in a general education school (*allgemeinbildende Schule* in German). Exclusion criteria were not fulfilling the inclusion criteria and not being able to operate a computer, laptop, tablet, or mobile phone.

#### Sample Size Considerations

To determine the minimum sample size, an a priori sample size calculation was conducted in G*Power (version 3.1.9.7) based on the following parameters: a small-to-medium effect size of η^2^_p_=0.025, an α level of .05, and a power of .90 for the interaction effect in a 2-factorial mixed ANOVA between the factors group and time point, with the knowledge quiz and self-reports (see the Measures section) as dependent variables. These were the primary hypotheses for this study. The analysis yielded a minimum total sample size of 412 participants per sample (eg, 206 individuals per group in each sample). Taking potential attrition into account, our aim was to recruit approximately 500 participants for each sample.

### Procedure

The study was conducted as a randomized controlled pre-post study with 2 measurements (T1, T2). Two independent samples and 2 groups per sample are relevant to this investigation. The trial was entirely web-based. The samples were students aged 14 years to 18 years and school teachers living in Germany. The market research institute was responsible for randomization. Randomization was performed independently for each sample after the participants initiated the study. A computer-based adaptive randomization technique without stratification was used, taking into account the current group sizes. The aim was to create groups of similar sizes. Participants were formally blinded to whether they were assigned to the control or experimental group, but there was relatively high face validity concerning the content of the intervention. Participants did not know which was the intervention of interest and which was the comparator.

After the initial data collection via web-based questionnaires at T1, participants from each sample were allocated to an experimental group or a control group in a randomized procedure. Each group was then shown an animated YouTube Health video (details are provided in the following sections). After watching the YouTube Health video, the participants completed a second set of questionnaires (T2). In total, data collection took about 20 minutes per participant, including watching the YouTube Health video and all measurements.

At T1, sociodemographic information was collected. In addition, the participants were presented with a stress-specific knowledge quiz and self-report questionnaire to assess mental health knowledge as well as a knowledge quiz and self-report questionnaire on fake news not relevant to this study. Results on these later questionnaires are not the focus of this publication but can be found in Multimedia Appendix 1, including Table S1. At T2, all quality measures were presented, and the knowledge quizzes and self-report questionnaires were repeated to assess any changes relative to T1.

### Materials and Measures

#### YouTube Health Videos

Two YouTube Health videos of approximately 2 minutes to 3 minutes in length were used in this study. The videos were presented to the participants on the same platform as the online questionnaires. The participants in the experimental group watched a YouTube Health video on stress to promote mental health literacy, and the participants in the control group watched a YouTube Health video on fake news with no focus on mental health literacy. Both YouTube Health videos are animated, show human protagonists, and provide central information on the corresponding topic with the help of a short entertaining storyline. The YouTube Health videos were produced by the independent nonprofit foundation Stiftung Gesundheitswissen and are publicly available on their website and YouTube channel. The experimental YouTube Health video designed to promote mental health literacy contains a general introduction on the topic of stress. It includes brief definitions of internal and external stressors, acute and chronic stress, and healthy and unhealthy coping mechanisms [40]. The control group YouTube Health video addresses the topic of fake news and covers the topics misinformation (unintentional) and disinformation (deceitful and intentional) in the contexts of click-baiting, satire, personal testimonials, deepfakes, and conspiracy theories [41]. A brief sound test was conducted before the YouTube Health videos were presented to ensure that all participants not only watched the videos but also listened to them. In the sound test, the participants heard a short audio and had to indicate what they heard in a multiple choice question (eg, dogs barking, bells ringing, piano music playing, or children laughing). Only if the correct answer was given, the survey continued, and the YouTube Health video was presented.

#### Measures

#### Sociodemographic Information

All participants reported their age and gender.

#### Knowledge Quiz

Before and after the video presentation, the participants completed 2 brief topic-specific knowledge quizzes. Crucially, these quizzes were answered by both groups; that is, all participants in the experimental and control groups answered items on both stress and fake news before and after watching their individual video. For each topic, a total of 16 items were presented in 4 blocks of 4 items. For each item, the participants had to indicate if the presented statement was correct or not. They could also choose an “I don’t know” option. For each correct response, 1 point was awarded to the participants. No points were awarded for any incorrect or “I don’t know” responses. Points were summed up separately across all items of each topic, and 2 topic-specific sum scores were calculated with a range of 0 to 16 points each. The items covered content presented in the videos (eg, on the definition of stress and stressors, coping mechanisms, and the distinction between chronic and acute stress for the mental health literacy video). For the fake news video, the items covered the topics click-baiting, deepfakes, misinformation, and disinformation. The items were constructed by the author team and pretested and discussed with experts involved in the production of the videos. The stress knowledge quiz is one of the two primary outcomes of this study, alongside the stress self-reports described in the following section. All other measures are secondary outcomes.

#### Self-Report

In addition, all participants answered 2 self-report measures on the topics of stress and fake news. Each measure consisted of 4 items answered on a 6-point Likert scale ranging from “strongly disagree” to “strongly agree.” Each item was a short statement referring to the ability of the respondent to explain certain concepts (eg, “I can explain how psychological stressors work” for the stress self-report and “I can explain how disinformation works” for the fake news self-report). A mean score was calculated for each topic, with a range of 1 to 6. The participants could also select an “I don’t know” option. All items in the knowledge quizzes and self-reports are in Tables S2-S5 in Multimedia Appendix 1.

#### Quality Measures

After watching the YouTube Health videos, the participants were asked to complete the Global Quality Scale (GQS), which consists of a single item. The participants were asked to evaluate the overall quality of the videos on a scale from 1 to 5, with higher values indicating higher overall quality [42]. A value of 4 or 5 can be considered as high quality [43]. The scale was initially developed for patients to evaluate videos containing health information [42]. The instructions were translated to German and slightly adjusted by replacing the word “patients” with “viewers.”

To assess the educational quality of the videos, the German version of the “learning and value” subscale of the Student Evaluation of Educational Quality (SEEQ) instrument was used at T2 [44,45]. The scale has 5 items rated on a 5-point Likert scale ranging from “strongly disagree” (1) to “strongly agree” (5). The instrument was initially developed to measure educational quality in higher education. The items were slightly adapted to apply to the evaluation of videos. A mean score was calculated across the 5 items, with higher values indicating higher educational quality.

Furthermore, the German version of the Visual Aesthetics of Websites Inventory (VisAWI) was used to assess the visual experience and aesthetics of the videos [46]. All 4 subscales, namely simplicity (5 items), diversity (5 items), colorfulness (4 items), and craftmanship (4 items), were presented. The items were slightly adjusted to apply to the evaluation of videos rather than websites, for which the instrument was originally developed. Each item is rated on a 7-point Likert scale ranging from “strongly disagree” to “strongly agree.” A mean score was calculated separately for each subscale, and an additional total mean score was calculated across all items. Higher mean scores denote higher quality, and scores greater than 4.5 indicate an overall positive evaluation [47].

#### Usability, Endorsement, and Motivation Items

In order to assess the potential usability of the YouTube Health videos in school settings and outside of it more thoroughly, all participants were asked whether they would watch explanatory videos of this kind in their free time, recommend them to friends, recommend them to relatives, and share them on social media, in 4 separate questions. Each of these endorsement questions was rated on a 4-point Likert scale with the options “no,” “likely no,” “likely yes,” and “yes.” Higher values denote a higher probability of watching, recommending, or sharing the video.

In addition, students were asked if it would motivate them if explanatory videos of this kind were used in (1) lessons, (2) in digital lessons, (3) as homework, (4) in project weeks, and (5) in class chats or digital classroom platforms and if it would motivate them if (6) their teacher recommended the videos to them. Teachers were asked if they would use these or similar videos in (1) lessons, (2) in digital lessons, (3) as homework, (4) in project weeks, and (5) in class chats or digital classroom platforms and whether they would recommend them to (6) teacher colleagues and (7) their students. These questions were also answered on a 4-point Likert scale with the options “no,” “likely no,” “likely yes,” and “yes.” For teachers, higher values denote a higher probability of using or recommending the videos. For students, higher values denote a higher probability of being motivated by these kinds of videos.

### Statistical Analysis

Data preprocessing was conducted with the statistical software SPSS (version 29.0.2.0; IBM Corp). All further statistical analyses were conducted with JASP (version 0.19.1). For the power analyses, G*Power (version 3.1.9.7) was used. The Cronbach α was calculated as a measure of internal consistency for all questionnaires with multiple items. The main analyses were separate 2-factorial ANOVAs with 1 between-participants factor (experimental group vs control group) and 1 repeated-measures factor (pre- and postassessment) for each dependent variable (ie, total score on knowledge quiz, mean score on the self-report measures) and each group (students, teachers). The primary hypotheses were tested with interaction effects between the groups and the repeated-measures factors. Significant interaction effects were followed by *t* tests for conditional comparisons: Separate *t* tests were calculated for the pre-post comparison of each group and for each group comparison at each measurement (pre, post). These post hoc *t* tests underwent Bonferroni correction, and corrected *P* values are reported. Effect sizes for the ANOVA main and interaction effects are reported as η^2^_p_. Effect sizes for the post hoc *t* tests are reported as Cohen *d*. For all analyses, α thresholds were set to .05, and *P* values are 2-sided. The secondary outcomes were only analyzed on a descriptive level.

## Results

### Sample Description

**Figure 1:**
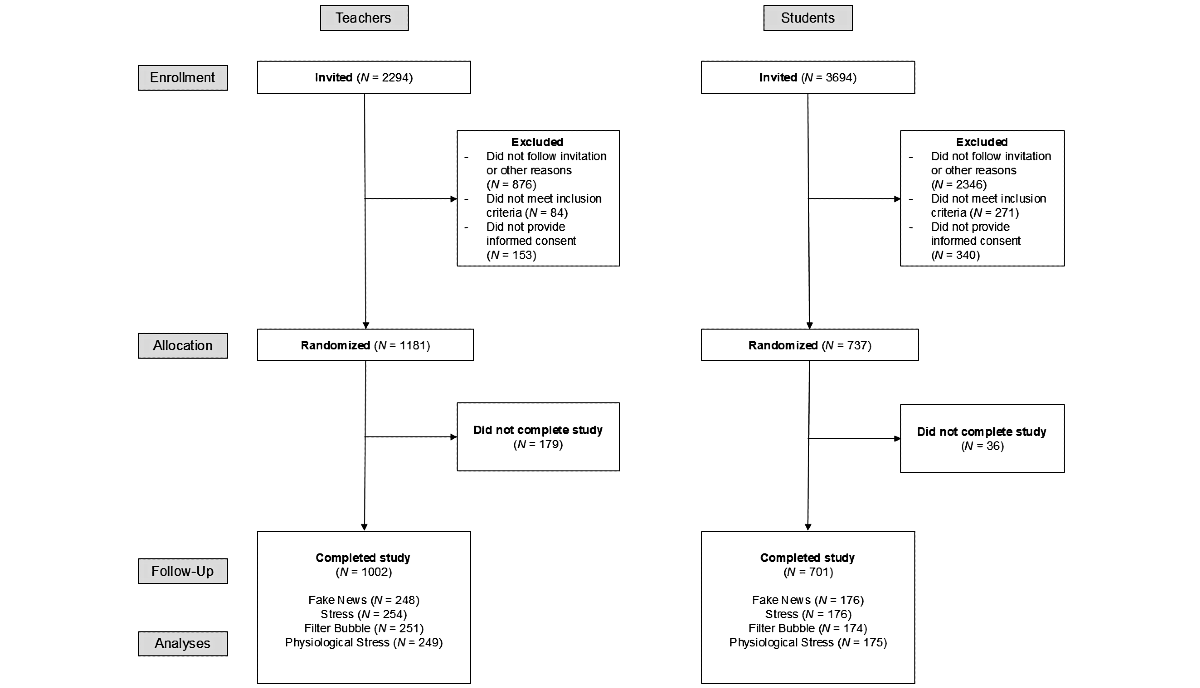
Participant flowchart.

We recruited and assigned 352 students and 502 teachers to the experimental and control groups using a randomized procedure. Detailed sample characteristics for each group can be found in Table 1. For the students, the targeted sample size of a minimum of 412 individuals could not be completed in the study period. Therefore, for this sample, post hoc analyses of achieved power for the primary analyses (eg, ANOVA interaction effects) were conducted and are reported with the respective results. Details on recruitment and attrition are in Figure 1 and Table S6 in Multimedia Appendix 1.

**Table 1:** Characteristics of the teachers and students.

Characteristics			Students					Teachers		
			Experimental group (n=176)		Control group (n=176)		Experimental group (n=254)			Control group (n=248)
Age (years), mean (SD)			15.55 (1.31)		15.44 (1.29)		50.48 (10.43)			51.32 (9.79)
**Gender, n (%)**										
	Men	91 (51.7)		84 (47.7)		96 (37.8)			99 (39.9)	
	Women	85 (48.3)		92 (52.3)		158 (62.2)			149 (60.1)	

### Quality of the Measures

Cronbach α indices of internal consistency for all scales can be found in Table 2.

**Table 2:** Cronbach α indices of internal consistency.

Scale			Students, α		Teachers, α
**Knowledge quiz**					
	T1^a^	0.44		0.22	
	T2^b^	0.63		0.64	
**Self-report questionnaire**					
	T1	0.88		0.93	
	T2	0.93		0.95	
**SEEQ^c^**					
	Learning and value	0.82		0.80	
**VisAWI^d^**					
	Overall	0.95		0.94	
	Simplicity	0.86		0.86	
	Diversity	0.83		0.82	
	Colorfulness	0.80		0.78	
	Craftsmanship	0.82		0.80	

^a^T1: baseline.

^b^T2: postintervention.

^c^SEEQ: Student Evaluation of Educational Quality.

^d^VisAWI: Visual Aesthetics of Websites Inventory.

### Knowledge Quizzes and Self-Report in the Students’ Sample

**Figure 2:**
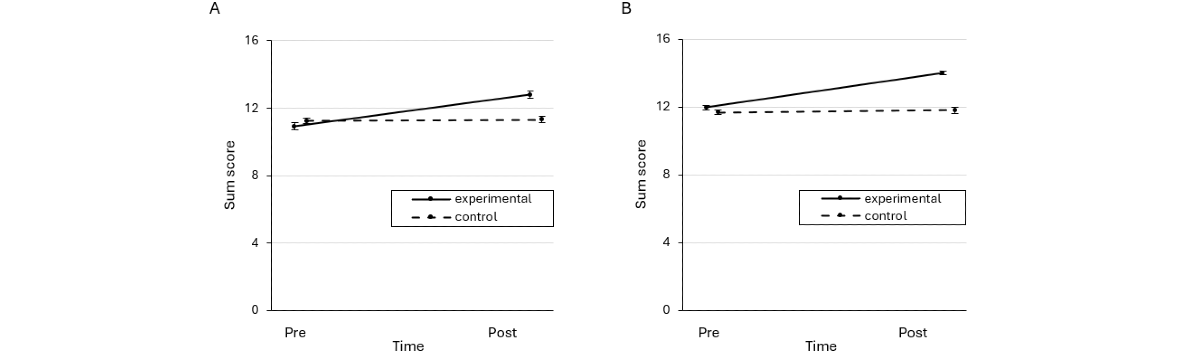
Mean sum scores for the objective knowledge tests in the experimental and control groups of (A) students and (B) teachers. Error bars represent the standard errors of the mean.

**Figure 3:**
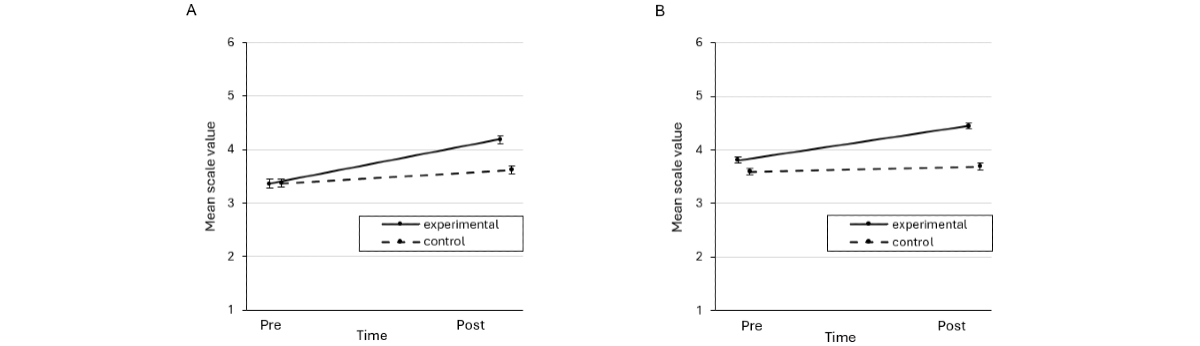
Mean scores for the knowledge self-reports in the experimental and control groups of (A) students and (B) teachers. Error bars represent the standard errors of the mean.

Detailed descriptive statistics for the stress-specific knowledge quizzes and self-report questionnaires for the students can be found in Tables 3 and 4 and Figures 2 and 3. There were significant main effects of time (*F*_1,350_=46.34, *P<*.001, η^2^_p_=0.117) and group (*F*_1,350_=6.05, *P=*.01, η^2^_p_=0.017) and a significant time×group interaction (*F*_1,350_=39.15, *P<*.001, η^2^_p_=0.101) on stress-specific knowledge. Post hoc *t* tests revealed a significant increase from T1 to T2 in the experimental group (*t*_175_=9.24, *P<*.001, *d*=0.71) but not in the control group (*t*_175_=0.39, *P=*.70, *d*=0.03). The experimental group had higher knowledge than the control group at T2 (*t*_350_=5.15, *P<*.001, *d*=0.57) but not at T1 (*t*_350_=−1.12, *P*=.26, *d*=−0.12). In addition, significant main effects of time (*F*_1,347_=103.65, *P*<.001, η^2^_p_=0.230) and group (*F*_1,347_=8.59, *P*=.004, η^2^_p_=0.024) and a significant time×group interaction (*F*_1,347_=29.11, *P*<.001, η^2^_p_=0.077) were observed for the stress self-report on subjective knowledge. Post hoc *t* tests revealed a significant increase from T1 to T2 in both the experimental group (*t*_173_=11.00, *P<*.001, *d*=0.81) and control group (*t*_174_=3.39, *P<*.001, *d*=0.25), with a higher effect size in the experimental group. The experimental group reported higher subjective knowledge than the control group at T2 (*t*_347_=5.30, *P*<.001, *d*=0.56) but not at T1 (*t*_347_=−0.07, *P*=.94, *d*=−0.01).

For the respective effect sizes of η^2^_p_=0.101 and η^2^_p_=0.077 and of the interaction effects, post hoc power analyses showed that power exceeded .99 in both analyses.

**Table 3:** Descriptive statistics and results of the group comparisons for the stress-specific knowledge quizzes and self-reports completed by the experimental and control groups of teachers and students.

Groups and timeframe				Experimental group, mean (SD)		Control group, mean (SD)		Group comparison, *t* test (*df*)		*P* value		Cohen *d*
**Students**												
	**Knowledge quiz^a^**											
		T1^b^	10.92 (2.90)		11.23 (2.16)		−1.12 (350)		.26		−0.12	
		T2^c^	12.81 (2.96)		11.31 (2.49)		5.15 (350)		<.001		0.57	
	**Self-report assessment^d^**											
		T1	3.36 (1.11)		3.37 (0.96)		−0.07 (347)		.94		−0.01	
		T2	4.19 (1.00)		3.62 (0.99)		5.30 (347)		<.001		0.56	
**Teachers**												
	**Knowledge quiz**											
		T1	11.97 (2.04)		11.67 (2.10)		1.62 (500)		.11		0.14	
		T2	14.01 (1.76)		11.81 (2.72)		10.80 (500)		<.001		1.01	
	**Self-report assessment**											
		T1	3.81 (0.94)		3.60 (0.98)		2.46 (500)		.01		0.23	
		T2	4.44 (0.77)		3.69 (1.00)		9.51 (500)		<.001		0.82	

^a^Possible score range: 0-16.

^b^T1: baseline.

^c^T2: postintervention.

^d^Possible score range: 1-6.

**Table 4:** Time effects for the stress-specific knowledge quizzes and self-reports completed by the experimental and control groups of teachers and students.

Groups and assessment type				Time effects between baseline (T1) and postintervention (T2)				
				*t* test (*df*)		*P* value		Cohen *d*
**Students**								
	**Knowledge quiz**							
		Experimental group	9.24 (175)		<.001		0.71	
		Control group	0.39 (175)		.70		0.03	
	**Self-report assessment**							
		Experimental group	11.00 (173)		<.001		0.81	
		Control group	3.39 (174)		<.001		0.25	
**Teachers**								
	**Knowledge quiz**							
		Experimental group	13.83 (253)		<.001		0.93	
		Control group	0.89 (247)		.37		0.06	
	**Self-report assessment**							
		Experimental group	13.34 (253)		<.001		0.69	
		Control group	1.92 (247)		.06		0.10	

### Knowledge Quizzes and Self-Report in the Teachers’ Sample

Detailed descriptive statistics for the stress-specific knowledge quizzes and self-reports for the teachers can be found in Tables 3 and 4 and Figures 2 and 3. There were significant main effects of time (*F*_1,500_=107.31, *P*<.001, η^2^_p_=0.177) and group (*F*_1,500_=58.07, *P*<.001, η^2^_p_=0.104) and a significant time×group interaction (*F*_1,500_=82.59, *P*<.001, η^2^_p_=0.142) on stress-specific knowledge. Post hoc *t* tests revealed a significant increase from T1 to T2 in the experimental group (*t*_253_=13.83, *P<*.001, *d*=0.93) but not in the control group (*t*_247_=0.89, *P=*.37, *d*=0.06). There were significant group differences at T2 (*t*_500_=10.80, *P<*.001, *d*=1.01) but not at T1 (*t*_500_=1.62, *P*=.11, *d*=0.14). In addition, significant main effects of time (*F*_1,500_=115.40, *P*<.001, η^2^_p_=0.188) and group (*F*_1,500_=41.16, *P*<.001, η^2^_p_=0.076) and a significant time×group interaction (*F*_1,500_=64.24, *P*<.001, η^2^_p_=0.114) were delineated for self-reported stress-specific knowledge. Post hoc *t* tests revealed a significant increase from T1 to T2 in the experimental group (*t*_253_=13.34, *P<*.001, *d*=0.69) but not the control group (*t*_247_=1.92, *P=*.06, *d*=0.10). There were significant group differences at T2 (*t*_500_=9.51, *P*<.001, *d*=0.82) as well as at T1 (*t*_500_=2.46, *P*=.01, *d*=0.23).

### Quality Measures

On average, both samples rated the overall quality (GQS), the educational quality (SEEQ), and the visual experience (VisAWI) of both videos as positive (details in Table 5). For the GQS, values ≥4 were achieved in both samples and for both videos, indicating high overall quality. Likewise, for the VisAWI, the cutoff of 4.5 was exceeded in all subscales and the overall score in both samples and for both videos.

**Table 5:** Descriptive statistics for the quality scales for teachers and students.

Quality scales		Students							Teachers				
		Experimental group			Control group			Experimental group				Control group	
		Mean (SD)	Range	Mean (SD)		Range	Mean (SD)			Range	Mean (SD)		Range
Overall GQS^a^		4.27 (0.68)	1-5	4.13 (0.68)		1-5	4.22 (0.57)			2-5	4.23 (0.65)		2-5
SEEQ^b^ learning and value		3.50 (0.82)	1-5	3.56 (0.78)		1.4-5	3.34 (0.80)			1-5	3.68 (0.79)		1-5
**VisAWI^c^**													
	Overall	5.07 (0.83)	3.1-7	5.01 (0.95)		1-7	5.39 (0.80)			2.9-7	5.17 (0.85)		2.2-7
	Simplicity	5.27 (0.89)	2.6-7	5.16 (0.95)		1-7	5.62 (0.83)			2.8-7	5.33 (0.93)		1-7
	Diversity	4.86 (0.92)	2.4-7	4.77 (1.07)		1-7	5.10 (0.92)			2.4-7	4.95 (0.96)		1.6-7
	Colorfulness	4.91 (0.96)	2.3-7	4.94 (1.07)		1-7	5.28 (0.94)			2.5-7	4.96 (0.97)		1.5-7
	Craftsmanship	5.22 (0.98)	3.3-7	5.19 (1.04)		1-7	5.59 (0.88)			3-7	5.45 (0.96)		1.8-7

^a^GQS: Global Quality Scale.

^b^SEEQ: Student Evaluation of Educational Quality.

^c^VisAWI: Visual Aesthetics of Websites Inventory.

### Usability, Endorsement, and Motivation Items

Descriptive statistics for the usability, endorsement, and motivation items can be found in Table 6 for the students and Table 7 for the teachers. Means and SDs for each item can be found in Tables S7-S9 in Multimedia Appendix 1.

Overall, most students reported that they would not watch videos of this kind in their free time nor share them on social media. However, most students would recommend them to their friends and relatives. Most students reported that they would feel motivated if videos of this kind were used in lessons or digital lessons, as homework, in class chats, or in project weeks or if they were recommended to them by their teachers.

Most teachers reported that they would watch videos of this kind in their free time and recommend them to friends, relatives, their teacher colleagues, and students but that they would not share them on social media. For use in schools, most teachers reported that they would use the videos in lessons and digital lessons, as homework, in project weeks, and in class chats or digital learning platforms.

**Table 6:** Responses to the endorsement and motivation items by students. The Cumulative percentages may exceed or fall below 100% due to rounding for the Control and the Experimental groups.

Questions			Control group, n (%)	Experimental group, n (%)
**Endorsement items**				
	**Would you watch explanatory videos of this kind in your free time?**			
		No / likely no	103 (58.5)	100 (56.8)
		Yes / likely yes	73 (41.5)	76 (43.2)
		Do not know / no answer	0 (0)	0 (0)
	**Would you recommend explanatory videos of this kind to your friends?**			
		No / likely no	80 (45.5)	84 (47.7)
		Yes / likely yes	96 (54.5)	92 (52.3)
		Do not know / no answer	0 (0)	0 (0)
	**Would you recommend explanatory videos of this kind to your relatives?**			
		No / likely no	74 (42)	74 (42)
		Yes / likely yes	102 (58)	102 (58)
		Do not know / no answer	0 (0)	0 (0)
	**Would you share explanatory videos of this kind on social media?**			
		No / likely no	112 (63.6)	129 (73.3)
		Yes / likely yes	64 (36.4)	47 (26.8)
		Do not know / no answer	0 (0)	0 (0)
**Motivation items**				
	**Would it motivate you if explanatory videos of this kind were used in lessons?**			
		No / likely no	36 (20.5)	32 (18.5)
		Yes / likely yes	140 (79.5)	143 (81.2)
		Do not know / no answer	0 (0)	1 (0.6)
	**Would it motivate you if explanatory videos of this kind were used in digital lessons?**			
		No / likely no	30 (17)	22 (12.5)
		Yes / likely yes	146 (83)	153 (86.9)
		Do not know / no answer	0 (0)	1 (0.6)
	**Would it motivate you if explanatory videos of this kind were used in the context of homework?**			
		No / likely no	45 (25.6)	35 (19.9)
		Yes / likely yes	131 (74.4)	140 (79.5)
		Do not know / no answer	0 (0)	1 (0.6)
	**Would it motivate you if explanatory videos of this kind were used in project weeks?**			
		No / likely no	35 (19.9)	27 (15.3)
		Yes/ likely yes	141 (80.1)	148 (84.1)
		Do not know / no answer	0 (0)	1 (0.6)
	**Would it motivate you if explanatory videos of this kind were shared in class chats or digital learning and teaching platforms?**			
		No / likely no	68 (38.6)	57 (32.4)
		Yes / likely yes	108 (61.4)	118 (67)
		Do not know / no answer	0 (0)	1 (0.6)
	**Would it motivate you if explanatory videos of this kind were recommended to you by your teachers?**			
		No / likely no	58 (33)	52 (29.6)
		Yes / likely yes	118 (67)	123 (69.8)
		Do not know / no answer	0 (0)	1 (0.6)

**Table 7:** Responses to the endorsement and motivation items by teachers. The Cumulative percentages may exceed or fall below 100% due to rounding for the Control and the Experimental groups.

Questions				Control group, n (%)		Experimental group, n (%)
**Endorsement items**						
	**Would you watch explanatory videos of this kind in your free time?**					
		No / likely no	91 (36.7)		90 (35.4)	
		Yes / likely yes	157 (63.3)		164 (64.5)	
		Do not know / no answer	0 (0)		0 (0)	
	**Would you recommend explanatory videos of this kind to your friends?**					
		No / likely no	81 (32.7)		102 (40.2)	
		Yes / likely yes	167 (67.3)		151 (59.5)	
		Do not know / no answer	0 (0)		1 (0.4)	
	**Would you recommend explanatory videos of this kind to your relatives?**					
		No / likely no	78 (31.5)		89 (35)	
		Yes / likely yes	170 (68.5)		164 (64.6)	
		Do not know / no answer	0 (0)		1 (0.4)	
	**Would you share explanatory videos of this kind on social media?**					
		No / likely no	170 (68.6)		178 (70.1)	
		Yes / likely yes	78 (31.4)		75 (29.5)	
		Do not know / no answer	0 (0)		1 (0.4)	
**Usability items**						
	**Would you use explanatory videos of this kind in your lessons?**					
		No / likely no	47 (19)		44 (17.3)	
		Yes / likely yes	201 (81)		210 (82.7)	
		Do not know / no answer	0 (0)		0 (0)	
	**Would you use explanatory videos of this kind in your digital lessons?**					
		No / likely no	38 (15.3)		47 (18.5)	
		Yes / likely yes	209 (84.3)		207 (81.5)	
		Do not know / no answer	1 (0.4)		0 (0)	
	**Would you use explanatory videos of this kind in the context of homework?**					
		No / likely no	87 (35.1)		97 (38.2)	
		Yes / likely yes	161 (64.9)		157 (61.8)	
		Do not know / no answer	0 (0)		0 (0)	
	**Would you use explanatory videos of this kind in project weeks?**					
		No / likely no	43 (17.3)		49 (19.3)	
		Yes / likely yes	205 (82.7)		205 (80.7)	
		Do not know / no answer	0 (0)		0 (0)	
	**Would you share explanatory videos of this kind in class chats or digital learning and teaching platforms?**					
		No / likely no	101 (40.7)		108 (42.5)	
		Yes / likely yes	146 (58.9)		146 (57.5)	
		Do not know / no answer	1 (0.4)		0 (0)	
	**Would you recommend explanatory videos of this kind to your teacher colleagues?**					
		No / likely no	63 (25.4)		85 (33.5)	
		Yes / likely yes	184 (74.2)		169 (66.5)	
		Do not know / no answer	1 (0.4)		0 (0)	
	**Would you recommend explanatory videos of this kind to your students?**					
		No / likely no	43 (17.3)		64 (25.2)	
		Yes / likely yes	204 (82.3)		190 (74.8)	
		Do not know / no answer	1 (0.4)		0 (0)	

## Discussion

### Study Summary

This study investigated the effectiveness of a short educational YouTube Health video for enhancing mental health knowledge as a facet of mental health literacy among adolescents and teachers and its usability in a school setting—a critical environment for mental health (literacy) promotion [9,10,28]. It was hypothesized that both students and teachers would report higher mental health knowledge after watching the video on mental health (experimental group) when compared with a control group who watched a video similar in style but regarding a different topic. In addition, the overall quality, educational quality, and visual appeal of the videos were investigated, and we assessed whether the videos were deemed suitable for use in and outside school settings.

### Key Findings and Implications

The study results revealed higher statistically significant increases in both objective knowledge (quiz scores) and subjective knowledge (self-report questionnaires) in the experimental groups than in the control groups of both students and teachers. These findings underline that videos can be effective tools for disseminating mental health information and improving aspects of mental health literacy [36] as has already been shown for other types of interventions [9,29,35]. Conversely, the control group watched a video on fake news, matched for length and visual style, which allowed the attribution of the observed knowledge increase to the mental health content rather than the general video format. At T1, there was a small group difference in self-reported knowledge in the teachers, with higher a priori knowledge in the experimental group than in the control group. This was most likely a random—and not systematic—variation as the participants were allocated to the 2 groups through randomization.

High ratings for overall quality, educational quality, and visual appeal across both samples showed that the YouTube Health videos’ design and content were well-received. For both teachers and students, established thresholds for high quality were exceeded, if available [43,47]. Furthermore, most students reported that they would be motivated if the presented or similar videos were used in lessons or digital lessons, as homework, in project weeks, or in class chats and if teachers recommended these videos to them. Similarly, most teachers indicated that they would use these and similar videos in various educational contexts and that they would recommend them to both their colleagues and students. This underlines the potential for integrating such resources into school curricula [9,10]. For use outside of school settings, most students reported that they would not likely watch these and similar videos in their free time nor share them on social media. However, many students also reported that they would recommend videos of this kind to friends and family.

Together, this pattern of results underscores the pivotal role of schools in providing access to mental health resources for adolescents. Schools and school authorities should leverage this unique position to implement evidence-based initiatives that enhance mental health literacy and promote mental health in general [9,10,29,48]. For example, YouTube Health videos such as the ones evaluated in this study could be used to prompt classroom discussions on the topic of mental health care. Implementation of these videos in school curricula should be complemented by the use of other resources such as topic-specific worksheets or e-learning courses [49,50]. In this context, adequate qualification and training of teachers who bear substantial responsibility for the implementation of mental health initiatives in daily school life should be given special emphasis [51]. Training programs should include components to increase mental health knowledge, reduce stigma, and foster confidence in teachers [51]. In addition, tight collaboration with other professionals and mental services should be promoted [10]. Importantly, mental health promotion in schools should be regarded not only as dealing with already existing mental health problems but also—and maybe more importantly—in terms of prevention of these problems and promotion of positive mental health and well-being [10].

Interestingly, on a descriptive level, the usability, endorsement, and motivation items were rated similarly for both the mental health and fake news videos. These findings highlight that the positive evaluation of the videos is most likely not related to their specific topic but to their design and style. Based on this, it may be concluded that the development and dissemination of videos in a similar style, and covering a wide range of topics, could be useful for implementation in schools to promote mental health literacy. The potential of uploading more high-quality content to public sources such as YouTube and making use of programs to promote science-based health content with programs like YouTube Health should therefore be fully exploited [37].

The observed effect sizes for the interaction effects and post hoc tests were medium to large [52], but the numerical increases on the sum scores were rather small (approximately 2 points in the quiz sum scores in both samples), which might have been due to a ceiling effect as participants from all groups already reached high quiz scores at T1, before the intervention. This finding has two implications: First, future evaluation studies could benefit from more difficult tests to ensure more variance, possibly safeguarded by more thorough piloting, and second, the finding speaks for already medium-to-high levels of mental health knowledge on the topic of stress before the intervention. It might be helpful to explore specific mental health knowledge gaps more in-depth to inform the development of more targeted videos in the future.

### Limitations

The study results presented here have a few limitations. First, the evaluation of the intervention’s effects relied partially on self-reports, and low Cronbach α indices of internal consistency were obtained for the quiz scores, indicating low reliability of the measures. This might be due to the small number of items that were constructed to cover a wide range of topics addressed in the video. Future evaluations should therefore include more items and conduct more thorough pretesting to ensure higher reliability. Additionally, validated and broader instruments should be used alongside self-constructed items to provide a more comprehensive assessment of mental health literacy. However, results were replicated in objective knowledge tests, which speaks for the validity of the self-reports. Second, there was only a very short follow-up period. Future investigations should test for the stability of the findings over longer periods of time. Third, the target sample size of 412 individuals was not reached for students. Of note, however, effect sizes were so large that high power was still achieved. Fourth, rather than being tested in a school setting, the intervention was tested online, which might come with disadvantages, such as a less controlled testing environment, potentially affecting the validity of the result [53]. To determine the generalizability of the findings, future studies should therefore also test the intervention in its intended primary setting of use, namely in school contexts. Last, only a single video on a specific topic was investigated and compared with a video similar in style but regarding a different topic. Our findings underline the feasibility of using short educational videos for the promotion of mental health literacy in schools. However, the scope of this promotion is limited by the video’s short length and simplicity. Therefore, we propose that videos of these types should be part of more comprehensive programs that combine various types of intervention methods such as other videos on the topic [36], e-learning courses [49,50], classroom discussions, and teaching materials [9] for the extensive promotion of mental health in school settings.

### Conclusion

The study findings highlight the feasibility of using short, educational YouTube Health videos as tools for enhancing mental health literacy in both students and their educators. The positive evaluation in terms of educational, aesthetic, and overall quality along with high levels of teachers’ and students’ endorsement and possible positive effects on students’ motivation are promising for developing and producing more videos of this kind and implementing them into school curricula. Overall, this evaluation is a first step toward the more widespread promotion of mental health literacy in schools to create a more supportive and informed school environment where mental health care is prioritized.

## Appendix

Multimedia Appendix 1 Assessment details and results.

Multimedia Appendix 2 CONSORT-eHealth (V 1.6.1).

## Abbreviations

DRKS: German Clinical Trial Register
GQS: Global Quality Scale
SEEQ: Student Evaluation of Educational Quality
VisAWI: Visual Aesthetics of Websites Inventory
WHO: World Health Organization

## Data Availability

The data sets generated and analyzed during this study are the property of the independent, nonprofit foundation Stiftung Gesundheitswissen and are available on reasonable request. Requests to access the data sets should be directed to lars.koenig@stiftung-gesundheitswissen.de.
