# Supplementary material for: Effects of YouTube Health Videos on Mental Health Literacy in Adolescents and Teachers: Randomized Controlled Trial
Source: JMIR Ment Health. 2025 Jul 31;12:e76004. doi: 10.2196/76004 (PMC12355139; doi:10.2196/76004)
Supplement: Multimedia Appendix 1 [file mental_v12i1e76004_app1.pdf]

Multimedia Appendix of Supplementary Files

Effects of YouTube Health Videos on Mental Health Literacy in Adolescents and Teachers: Randomized Controlled Trial

Rebekka Schröder[1], Tim Hamer[1], Victoria Kruzewitz[1], Ellen Busch[1], Ralf Suhr[1], Lars König[1]

[1]Stiftung Gesundheitswissen, Berlin, Germany

* Corresponding author:
Dr. rer. nat. Lars König
lars.koenig@stiftung-gesundheitswissen.de
+49 30 4195492-62

Knowledge quizzes and self-reports in the students' sample

Descriptive statistics for the fake news knowledge quizzes and self-reports for the students' sample can be found in Table S1. There were significant main effects of time (F(1,350) = 47.49, p < .001, η^2_p = .119), group (F(1,350) = 15.74, p = .001, η^2_p = .043) and a significant time × group interaction (F(1,350) = 68.39, p < .001, η^2_p = .163) on fake news knowledge. Post-hoc t-tests revealed a significant increase from T1 to T2 in the fake news video group (t(175) = 10.72, p < .001, d = 0.75), but not in the stress video group (t(175) = −0.98, p = .330, d = −0.07). The fake news video group had higher knowledge than the stress group at T2 (t(350) = 6.94, p < .001, d = 0.78), but not at T1 (t(350) = −0.34, p = .733, d = −0.03). In addition, significant main effects of time (F(1,299) = 40.67, p < .001, η^2_p = .120), group (F(1,299) = 4.39, p = .037, η^2_p = .014) and a significant time × group interaction (F(1,299) = 15.18, p < .001, η^2_p = .048) were observed for the self-report on subjective knowledge about fake news. Post-hoc t-tests revealed a significant increase from T1 to T2 in the fake news video group (t(148) = 7.23, p < .001, d = 0.52), but not the stress video group (t(151) = 1.76, p = .079, d = 0.13). The fake news video group reported higher subjective knowledge than the stress video group at T2 (t(299) = 3.83, p < .001, d = 0.42), but not at T1 (t(299) = 0.16, p = .876, d = 0.02).

Knowledge quizzes and self-reports in the teachers' sample

Descriptive statistics for the fake news knowledge quizzes and self-reports for the teachers' sample can be found in Table S1. There were significant main effects of time (F(1,500) = 183.17, p < .001, η^2_p = .268), group (F(1,500) = 49.89, p < .001, η^2_p = .091) and a significant time × group interaction (F(1,500) = 184.85, p < .001, η^2_p = .270) on fake news knowledge. Post-hoc t-tests revealed a significant increase from T1 to T2 in the fake news video group (t(247) = 19.07, p < .001, d = 1.05), but not in the stress video group (t(253) = −0.04, p = .965, d = −0.00). There were significant group differences at T2 (t(500) = 12.06, p < .001, d = 1.10), but not at T1 (t(500) = 0.46, p = .643, d = 0.04). In addition, a significant main effect of time (F(1,412) = 116.68, p < .001, η^2_p = .221) and a significant time × group interaction (F(1,412) = 33.77, p < .001, η^2_p = .076) but no main effect of group (F(1,412) = 2.60, p = .108, η^2_p = .006) were delineated for the self-reported knowledge on fake news. Post-hoc t-tests revealed a significant increase from T1 to T2 in the fake news video group (t(210) = 11.86, p < .001, d = 0.60), and the stress video group (t(202) = 3.50, p < .001, d = 0.18), with a larger effect size in the fake news video group. There were significant group differences at T2 (t(412) = 3.66, p < .001, d = 0.36), but not at T1 (t(412) = −0.63, p = .529, d = −0.06).

Table S1: Descriptive statistics (means and standard deviations) in the fake news knowledge quizzes and self-reports of the experimental groups in the teachers' and students' samples.

	**Fake news video group**		**Stress video group**	
	**T1**<br>*M (SD)*	**T2**<br>*M (SD)*	**T1**<br>*M (SD)*	**T2**<br>*M (SD)*
**Students**				
Knowledge quiz	8.96 (3.29)	11.59 (3.46)	9.08 (3.28)	8.84 (3.94)
Self-report	4.03 (0.92)	4.50 (0.79)	4.02 (0.98)	4.13 (0.90)
**Teachers**				
Knowledge quiz	9.13 (3.22)	12.57 (2.76)	9.00 (3.21)	8.99 (3.80)
Self-report	4.01 (0.94)	4.58 (0.94)	4.06 (0.98)	4.24 (0.95)

Note: Means (*M*) and standard deviations (*SD*). Possible ranges 0–16 for the knowledge quiz and 1–6 for the self-report.

Table S2: Items of the stress-specific knowledge quizzes (in German, English translation in brackets).

1	**Wann entsteht typischerweise Stress?**<br>*(When does stress typically occur?)*	**Correct response**
	Wenn innere Reize als Belastung wahrgenommen werden.<br>*(When internal stimuli are perceived as a burden.)*	✓
	Wenn innere Reize nicht ausreichend wahrgenommen werden.<br>*(When internal stimuli are not sufficiently perceived.)*	
	Wenn innere Reize und äußere Reize nicht übereinstimmen.<br>*(When internal stimuli and external stimuli do not match.)*	
	Wenn äußere Reize als Belastung wahrgenommen werden.<br>*(When external stimuli are perceived as a burden.)*	✓
2	**Welche der folgenden Phänomene/Ereignisse können Stressoren darstellen?**<br>*(Which of the following phenomena/events can be stressors?)*	**Correct response**
	Zeitdruck<br>*(Time pressure)*	✓
	Krankheit<br>*(Illness)*	✓
	Todesfall<br>*(Fatality)*	✓
	Heiratsantrag<br>*(Marriage proposal)*	✓
3	**Welche zwei Arten von Stress werden typischerweise unterschieden?**<br>*(What types of stress are typically differentiated?)*	**Correct response**
	Akuter Stress<br>*(Acute stress)*	✓
	Bewältigungsstress<br>*(Coping stress)*	
	Chronischer Stress<br>*(Chronic stress)*	✓
	Vermeidungsstress<br>*(Avoidance stress)*	
4	**Welche der folgenden Aussagen zum Thema Stress sind korrekt?**<br>**(Which of the following statements about stress are correct?)**	**Correct response**
	Stress wird empfunden, wenn die persönliche Bewältigungsgrenze erreicht ist.<br>*(Stress is experienced when the personal coping capacity is reached.)*	✓
	Der Gedanke an einen Stressor kann ausreichen, um eine Stressreaktion auszulösen.<br>*(The thought of a stressor can be enough to trigger a stress reaction.)*	✓
	Gesundheitsschädigende Mittel können zur Stressbewältigung eingesetzt werden.<br>*(Harmful substances can be used to cope with stress.)*	✓
	Die Entfernung eines Stressauslösers ist eine Methode zur Stressbewältigung.<br>*(Removing a stress trigger is one method of coping with stress.)*	✓

Table S3: Items of the stress-specific self-reports (in German, English translation in brackets).

No.	Item
**1**	Ich kann die Funktionsweise von psychischen Stressoren erklären.<br>*(I can explain how psychological stressors work.)*
**2**	Ich kann die Funktionsweise von körperlichen Stressoren erklären.<br>*(I can explain how physical stressors work.)*
**3**	Ich kann die Funktionsweise von akutem Stress erklären.<br>*(I can explain how acute stress works.)*
**4**	Ich kann die Funktionsweise von chronischem Stress erklären.<br>*(I can explain how chronic stress works.)*

Note: These items were answered on a 6-point Likert scale ranging from 'strongly disagree' to 'strongly agree'.

Table S4: Items of the fake news-specific knowledge quizzes (in German, English translation in brackets).

**1**	**Welche der folgenden Aussagen zum Thema Fehlinformationen sind korrekt?** *(Which of the following statements on the subject of misinformation are correct?)*	**Correct response**
	Fehlinformationen bezeichnen Informationen, die inhaltlich falsch sind. *(Misinformation refers to information that is incorrect in terms of content.)*	✓
	Fehlinformationen bezeichnen Informationen, die mit einer Täuschungsabsicht erstellt wurden. *(Misinformation refers to information that was created with the intention to deceive.)*	
	Zeitungsenten sind ein Beispiel für Fehlinformationen. *(Newspaper hoaxes are an example of misinformation.)*	✓
	Fotomontagen sind ein Beispiel für Fehlinformationen. *(Photomontages are an example of misinformation.)*	
**2**	**Welche der folgenden Aussagen zum Thema Desinformationen sind korrekt?** *(Which of the following statements about disinformation are correct?)*	**Correct response**
	Desinformationen bezeichnen Informationen, die inhaltlich falsch sind. *(Disinformation refers to information that is incorrect in content.)*	✓
	Desinformationen bezeichnen Informationen, die mit einer Täuschungsabsicht erstellt wurden. *(Disinformation refers to information that was created with the intention to deceive.)*	✓
	Satire ist ein Beispiel für Desinformationen. *(Satire is an example of disinformation.)*	
	Deepfakes sind ein Beispiel für Desinformationen. *(Deepfakes are an example of disinformation.)*	✓
**3**	**Welche der folgenden Aussagen zum Thema Clickbaiting sind korrekt?** *(Which of the following statements on the subject of clickbaiting are correct?)*	**Correct response**
	Beim Clickbaiting werden oftmals irreführende Überschriften verwendet. *(Misleading headlines are often used in clickbaiting.)*	✓
	Clickbaiting verfolgt das Ziel, den Klickimpuls zu unterdrücken. *(The aim of clickbaiting is to suppress the click impulse.)*	
	Clickbaiting kann zur Kategorie der Fehlinformationen gezählt werden. *(Clickbaiting can be categorized as misinformation.)*	✓
	Clickbaiting kommt öfter auf Smartphones zur Anwendung als auf Laptops. *(Clickbaiting is used more often on smartphones than on laptops.)*	
**4**	**Wie nennt man das Phänomen, bei dem per künstlicher Intelligenz Bilder, Videos oder Audioaufnahmen erstellt werden, die es in der Realität nie gegeben hat?** *(What do you call the phenomenon in which artificial intelligence is used to create images, videos or audio recordings that never existed in reality?)*	**Correct response**
	FakeAIs *(FakeAIs)*	
	Digiscams *(Digiscams)*	
	Deepfakes *(Deepfakes)*	✓
	Mitigations *(Mitigations)*	

Table S5: Items of the fake news-specific self-reports (in German, English translation in brackets).

No.	Item
**1**	Ich kann die Funktionsweise von Fake News erklären.<br>*(I can explain how fake news work.)*
**2**	Ich kann die Funktionsweise von Fehlinformationen erklären.<br>*(I can explain how misinformation works.)*
**3**	Ich kann die Funktionsweise von Desinformationen erklären.<br>*(I can explain how disinformation works.)*
**4**	Ich kann die Funktionsweise von Deepfakes erklären.<br>*(I can explain how deepfakes work.)*

Note: These items were answered on a 6-point Likert scale ranging from 'strongly disagree' to 'strongly agree'.

Table S6: Recruitment and participation details in each sample.

	Students' sample	Teachers' sample
**Number of individuals invited to the study**	3694	2294
**Number of individuals who did not meet the screening criteria**	271	84
**Number of individuals without consent**	340	153
**Number of individuals who started but did not finish the study**	36	179
**Number of individuals with complete interviews**	701	1002

Note: These total numbers refer to the larger study with a total of four groups per sample, of which only two groups (stress video group, fake news video group) are relevant to the present study. Refer to the *Ethical considerations* section of the main manuscript for more details. Invitation numbers in the students' sample refer to the number of potential parents invited from the panel (details in the *Recruitment and data collection* section in the main manuscript).

Table S7: Descriptive statistics for endorsement items in the teachers' and students' samples.

**Would you…**	**Students**		**Teachers**	
	**Stress video group** *M (SD)*	**Fake news video group** *M (SD)*	**Stress video group** *M (SD)*	**Fake news video group** *M (SD)*
… watch explanatory videos of this kind in your free time?	2.33 (0.89)	2.29 (0.91)	2.69 (0.90)	2.65 (0.91)
… recommend explanatory videos of this kind to your friends?	2.50 (0.88)	2.48 (0.96)	2.66 (0.91)	2.72 (0.94)
… recommend explanatory videos of this kind to your relatives?	2.61 (0.91)	2.61 (0.96)	2.72 (0.91)	2.75 (0.94)
… share explanatory videos of this kind on social media?	2.05 (0.90)	2.11 (0.96)	2.00 (0.99)	2.04 (0.99)

Note: Range 1–4 for all groups and items.

Table S8: Descriptive statistics for motivation scales in the students' sample.

**Would it motivate you if explanatory videos of this kind were…**	**Stress video group** *M (SD)*	**Fake news video group** *M (SD)*
… used in lessons?	3.11 (0.79)	3.02 (0.80)
… used in digital lessons?	3.18 (0.73)	3.06 (0.79)
… used in the context of homework?	2.98 (0.80)	2.93 (0.87)
… used in project weeks?	3.10 (0.82)	3.02 (0.83)
… shared in class chats or digital learning and teaching platforms?	2.77 (0.91)	2.72 (0.91)
… recommended to you by your teachers?	2.91 (0.90)	2.85 (0.88)

Note: Range 1–4 for all groups and items.

Table S9: Descriptive statistics for the usability items in the teachers' sample.

**Would you …**	**Stress video group** *M (SD)*	**Fake news video group** *M (SD)*
… use explanatory videos of this kind in your lessons?	3.14 (0.83)	3.12 (0.87)
… use explanatory videos of this kind in your digital lessons?	3.16 (0.88)	3.24 (0.85)
… use explanatory videos of this kind in the context of homework?	2.73 (1.00)	2.83 (0.95)
… use explanatory videos of this kind in project weeks?	3.08 (0.89)	3.08 (0.83)
… share explanatory videos of this kind in class chats or digital learning and teaching platforms?	2.57 (1.04)	2.65 (1.06)
… recommend explanatory videos of this kind to your teacher colleagues?	2.74 (0.93)	2.96 (0.86)
… recommend explanatory videos of this kind to your students?	2.93 (0.88)	3.11 (0.81)

Note: Range 1–4 for all groups and items.
